# Blood platelets and sepsis pathophysiology: A new therapeutic prospect in critical ill patients?

**DOI:** 10.1186/s13613-017-0337-7

**Published:** 2017-12-01

**Authors:** Antoine Dewitte, Sébastien Lepreux, Julien Villeneuve, Claire Rigothier, Christian Combe, Alexandre Ouattara, Jean Ripoche

**Affiliations:** 1 INSERM U1026, BioTis, Univ. Bordeaux, 33000 Bordeaux, France; 2 Department of Anaesthesia and Critical Care II, Magellan Medico-Surgical Center, CHU Bordeaux, 33000 Bordeaux, France; 3 Department of Pathology, CHU Bordeaux, 33000 Bordeaux, France; 4 Cell and Developmental Biology Department, Centre for Genomic Regulation, The Barcelona Institute for Science and Technology, 08003 Barcelona, Spain; 5 Department of Nephrology, Transplantation and Haemodialysis, CHU Bordeaux, 33000 Bordeaux, France; 6 INSERM U1034, Biology of Cardiovascular Diseases, Univ. Bordeaux, 33600 Pessac, France

**Keywords:** Platelets, Sepsis, Inflammation, Intensive care

## Abstract

Beyond haemostasis, platelets have emerged as versatile effectors of the immune response. The contribution of platelets in inflammation, tissue integrity and defence against infections has considerably widened the spectrum of their role in health and disease. Here, we propose a narrative review that first describes these new platelet attributes. We then examine their relevance to microcirculatory alterations in multi-organ dysfunction, a major sepsis complication. Rapid progresses that are made on the knowledge of novel platelet functions should improve the understanding of thrombocytopenia, a common condition and a predictor of adverse outcome in sepsis, and may provide potential avenues for management and therapy.

## Background

Sepsis is a syndrome based on a dysregulated immune response to infection also involving non-immunologic mechanisms, including neuroendocrine, cardiovascular and metabolic pathways [1–3]. Due to its prevalence and high mortality rate, sepsis is a major public health issue [4, 5]. The contribution of blood platelets to sepsis pathophysiology has been the subject of renewed attention. First, alterations of platelet count are commonly encountered in the intensive care unit (ICU). Using common platelet counts thresholds, thrombocytopenia accounts for 20–50% of patients for the whole part of intensive care settings [6–9]. Thrombocytopenia or the non-resolution of thrombocytopenia is associated with poor outcome [8, 10–15]. Second, platelets are well-known players in coagulation and likely to contribute to disseminated intravascular coagulation (DIC). Third, beyond the confines of haemostasis and thrombosis, platelets are now acknowledged as essential actors of the immune response, reacting to infection and disturbed tissue integrity and contributing to inflammation, pathogen killing and tissue repair [16–21]. These advances in platelet biology have opened perspectives on the knowledge of sepsis pathophysiology and on its management. The matter is a complex one as platelets are not only vectors of inflammation contributing to vascular and tissue injury in acute or chronic inflammation [18, 22, 23], but also play an important role in the resolution of inflammation, vascular protection and the repair of damaged tissues. The friend and foe dialogue between platelets and endothelium has been extensively studied and is thought to be relevant to sepsis complications. Here we examine this enlarged spectrum of platelet functions and their relevance to the pathophysiology of multi-organ dysfunction (MOD) and discuss some potential links between these advances and sepsis management.

## Sepsis as a dysregulated host response to infection

Recent definition of sepsis [24] emphasizes the non-homoeostatic host response to infection that drives life-threatening organ dysfunctions. Activation of innate immune responses in sepsis realizes a systemic inflammatory condition. The inflammatory phase is characterized by the production of pro-inflammatory mediators and immune cell activation [25–29], and sepsis prognosis is linked to the magnitude and duration of this inflammatory response, high circulating cytokine levels being, for example, associated with poor outcome [30–32]. The triggering of innate immune responses by pathogens and pathogen-associated molecular patterns (PAMPs) has been identified as an early and primary mechanism [2, 31, 33–36]. Interestingly, mechanisms of non-septic systemic-associated inflammatory response syndrome (SIRS) as met in major surgery, severe trauma, extensive burns or pancreatitis may share common features with sepsis-associated SIRS, taking the form of a comparable early inflammatory storm that is triggered by alarmins released by damaged tissues [37]. However, the role played by this hyper-inflammatory phase in the progression of sepsis and its prognostic is to be understood in the context of an accompanying anti-inflammatory response and immunosuppression state, and much effort is made in elaborating a coherent vision of these opposite and complex events [38–43].

## Platelets: multifunctional tiny cytoplasmic fragments

Platelets are small (2–4 μm), anucleate, discoid-shaped cytoplasmic fragments released in the bloodstream during the fragmentation of polyploid megakaryocytes in bone marrow sinusoidal blood vessels [44]. In humans, a regulated steady platelet supply and clearance maintains numbers of 150,000–400,000 platelets per microlitre of blood. Platelet production is critically dependent on thrombopoietin (TPO) that acts for an important part on megakaryocyte progenitor proliferation/differentiation and on megakaryocyte maturation [45]. Platelets have a short lifespan, of up to 10 days. They are cleared out from the circulation by mechanisms involving lectin–carbohydrate recognition by splenic and liver macrophages and hepatocytes [46, 47].

Platelets harbour a large variety of mediators stored in a pool of morphologically distinct granules [48]. Granule cargo loading is carried out in megakaryocytes. Platelets also transport mediators, such as serotonin, that they uptake from plasma and can deliver at sites of activation. The cataloguing of platelet-derived mediators reflects the remarkable versatility of platelets in haemostasis, thrombosis and immune responses [49, 50].

The secretion of granule content following platelet activation by agonists is central to platelet functions. Platelet activation induces the expression of membrane proteins and the release of mediators via several mechanisms. Many of these mediators are preformed and stored in granules such as cytokines/chemokines and coagulation factors, others can be synthesized by translational pathways, such as IL-1β, and others are released by yet incompletely defined mechanisms such as CD154. Activated platelets also release vesicles, which include platelet microparticles (PMPs) and exosomes [51]. Platelets represent a major source of circulating MPs [52].

In pathological conditions associated with platelet activation, multiple agonists are generated. In fact, apart from classical strong agonists such as thrombin or collagen, there is an expanding list of agonists that can contribute to platelet activation. These additional platelet agonists have allowed a re-appreciation of mechanisms and role of platelet activation in vascular inflammation and thrombotic events associated with a range of infectious and inflammatory conditions [53].

The archetypal function of platelets is haemostasis. Platelets encounter inhibitory signals that prevent their activation in the healthy vasculature, such as nitric oxide and prostacyclin, which are released by endothelial cells (ECs). Platelets circulate in close proximity to the vessel wall, and the disruption of EC lining overcomes inhibitory signals and drives platelet adherence, activation and aggregation, which temporarily plug the damaged vessel. In this process, platelets also activate and confine coagulation at site of damage, particularly via the exposure of an efficient catalytic phospholipidic surface [54].

**Fig. 1:**
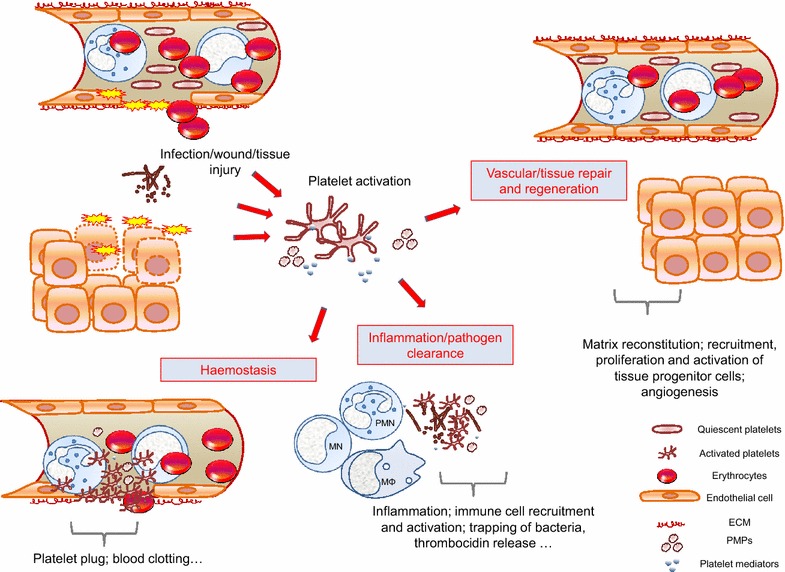
Platelets are integral players in the immune response, linking haemostasis, thrombosis, inflammation, pathogen clearance and tissue repair: a schematic representation. A growing body of evidence highlights a role for platelets beyond the confines of haemostasis and thrombosis. Some of platelet interfaces in innate immune response are schematized. Platelets are activated at sites of infection/tissue injury. Platelets and platelet-derived mediators contribute to arrest bleeding, to clear pathogens directly or indirectly by acting on various steps of the immune response, and to drive vascular/tissue repair by providing matrix building blocks and a multiplicity of signals that remodel matrix, attracting tissue progenitor cells and reconstructing the vascular frame. In doing so, platelets provide a coherent biological response contributing to cure infection and re-establish tissue architecture and homoeostasis. Scales are arbitrary. Platelet-derived microparticles (PMPs) recapitulate several of activated platelet functions. *ECM* extracellular matrix, *MN* monocytes, *PMN* polymorphonuclear neutrophils, *MΦ* macrophages

**Fig. 2:**
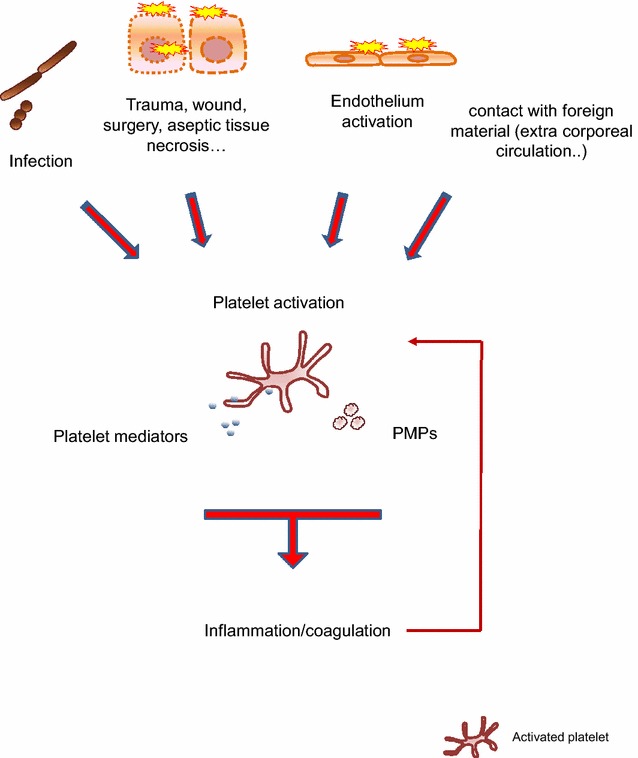
Platelets monitor and are activated in response to noxious signals. Platelets sense and are activated by multiple signals generated in danger situations met by the organism. Interaction with pathogens, endothelial cell/tissue injury and interaction with foreign material activate platelets (see text for details). Platelet activation sparks off a broad range of responses, including the activation of various inflammation and coagulation pathways. Signals generated in inflammation and coagulation can in return activate platelets (thin arrow). *PMPs* platelet microparticles

Besides binding to damaged vessels and preventing bleeding, platelets support a large spectrum of more recently studied functions, as could be reflected by the diversity of platelet mediators [55–57]. Platelets are activated in conditions that disrupt tissue homoeostasis and exert, directly and indirectly, a complex control over the different stages of inflammation, contributing to pathogen clearance, wound repair and tissue regeneration (Figs. 1, 2). As such, platelets are now acknowledged as essential components of the innate immune response, monitoring and rapidly responding to noxious signals.

## Platelets as key players in the inflammatory reaction; critical links with coagulation

Activated platelets secrete a profusion of pro-inflammatory material, cytokines/chemokines, vasoactive amines, eicosanoids, and components of proteolytic cascades that directly or indirectly, through the activation of bystander target cells, fuel inflammation [23, 58, 59]. ECs and leucocytes are prime targets for platelets. Endothelium is a non-adhesive, non-thrombogenic surface in normal conditions; when stimulated by inflammatory mediators, ECs undergo profound changes, collectively designed as “EC activation”, which include the expression of cell adhesion molecules and tissue factor, production of von Willebrand factor, cytokines/chemokines, proteases and vasoactive substances such as nitric oxide. Platelets adhere to activated ECs, following a multi-step process in which glycans play a critical role [60–62]. Inflammation can also alter the protective EC glycocalyx barrier, favouring platelet adhesion [63, 64]. During the adhesion process, platelets can be activated and in turn activate ECs. Platelet activation in inflammation can alter the vascular tone and lead to deleterious effects on vasculature integrity, by increasing vascular barrier permeability and contributing to the generation of cytopathic signals, for example by mediating reactive oxygen species generation by neutrophils [65]; these effects have to be paralleled with the opposed protective role of platelets (below) [66–69]. Leucocytes are a second critical target for platelets, the platelet/leucocyte dialogue being essential in inflammation; here, we focus on neutrophils and monocytes. Platelet/leucocyte interactions are a critical step in leucocyte recruitment, activation and migration in inflammation [70]. Platelet/neutrophil or platelet/monocyte interactions can occur at the EC surface, in clot/thrombi and in circulating blood [18, 70, 71], and platelets direct neutrophil/monocyte migration to sites of tissue injury [72, 73]. Moreover, platelets activate neutrophils and monocytes upon interaction, via several mechanisms, including the triggering of TREM-1 on neutrophils, leading to various pro-inflammatory responses [65, 74–77]. The formation of platelet/leucocyte aggregates in blood depends on platelet activation and is an early phenomenon in sepsis progression. For example, platelet/neutrophils complexes are elevated at early phases, while being reduced in complicated sepsis possibly reflecting peripheral sequestration or sepsis-associated thrombocytopenia [78, 79], and endotoxin administration in humans leads to an increased circulating platelet/neutrophil aggregates that follows a brief decrease [80]. Amplification of inflammation results from the reciprocal activation between platelets and their target cells [66], and circulating monocyte/and neutrophil/platelet aggregates may contribute to disseminate inflammatory signals [81]. Platelets also link several inflammatory cascades; for example, they propagate the activation of the complement system [82]. Commonly, cytokines have an induced expression that is regulated at the transcriptional/translational level. Most of platelet-derived inflammatory mediators are very rapidly released from activated platelets, making platelets instant providers of pro-inflammatory material. Cytokine bioactivity at organs remote from their source is debated as cytokine bioactivity may be hampered in plasma. Platelet transport may protect inflammatory mediators from otherwise degradation. Therefore, platelets play a central role in the inflammatory reaction. Importantly, they also contribute to the control and resolution of inflammation via several mechanisms, including the release of anti-inflammatory cytokines and inflammation pro-resolving mediators [83].

The activation of coagulation and inflammation cascades are consequences of platelet activation, and inflammation and coagulation pathways crosstalk [84]. For example, some platelet mediators have both inflammatory and pro-coagulant properties, such as polyphosphates [85]. Pro-inflammatory cytokines released by platelets can also activate the coagulation cascade at various steps [86]. Conversely, the activation of coagulation by platelets generates a number of inflammatory effectors, such as thrombin. Further, inflammatory mediators can impair anticoagulant and fibrinolysis pathway mechanisms, which may contribute to coagulation dysregulation in sepsis [87–89]. Platelet inflammatory mediators may thus contribute to sepsis coagulopathy [88–91]. DIC is a frequent and major complication of sepsis [41], and various mechanisms concur to involve platelets in DIC; only some can be mentioned. First, platelets support the generation of thrombin. Second, platelet links inflammation and coagulation. Third, platelets are major inducers of the release of pro-thrombotic scaffolds neutrophil extracellular traps (NETs) [92–96].

Notwithstanding, the involvement of PMPs in vascular inflammation and inflammatory disorders, including sepsis, has been emphasized [97–102]. PMPs retain many pro-inflammatory and pro-coagulant features of parent platelets and are thought to disseminate inflammatory and coagulation signals. Although they represent potential pathophysiological players in inflammatory disorders [52, 99, 100, 103, 104], their role in sepsis remains ill-understood.

## Platelets in vascular and tissue integrity

In normal wound healing, platelets establish regulatory crosstalks between soluble and cellular actors of tissue repair that concur to the various phases of inflammation and reestablishment of tissue homoeostasis [50, 83, 104, 105]. Platelets accumulate early, are activated at sites of tissue injury and intervene at each stage of tissue repair, the inflammatory, new tissue formation and remodelling stages. In fact, platelet-healing properties are already translated to the clinics [50, 55]. The best studied role of platelets in tissue homoeostasis is the preservation of resting and injured endothelium integrity, a critical point in MOD pathophysiology [71, 106–108]. The importance of platelets is exemplified by the disruption of the endothelium barrier associated with thrombocytopenia [109]. How platelets contribute is incompletely understood. Mechanisms include gap filling, production of EC mitogenic factors and factors enhancing the vascular barrier [71, 107]. On injured endothelium, platelets adhere to the vascular wall at sites of damage and immediate proximity, a first step in a sequence of events that lead to the initiation and the propagation of haemostasis, thrombosis and bleeding arrest [110, 111]. Platelets provide material for endothelium repair, including EC growth-promoting, antiapoptotic mediators, and attractants for progenitor cells endowed with vascular healing properties [104]. They help restoring the disrupted vascular network, providing positive and negative regulators of angiogenesis and stimulating angiogenic mediator production by target cells. Platelets are also important contributors to extracellular matrix (ECM) repair as they are a rich source of ECM components, ECM remodelling proteins, and fibro-competent cell activators. Platelets have however been found to both promote and prevent vascular permeability in inflammation. The differential regulation of vascular permeability by platelets has been studied for a large part in acute lung injury (ALI) models and will be presented in the corresponding section. Importantly, platelets are highly efficient at preventing bleeding in an inflammatory context [76, 107, 112]. The platelet count threshold needed for vasculoprotection in humans in normal or inflammatory conditions is an important question that remains to be answered [107]. More generally, platelets may play a broader role in organ regeneration. Platelets not only prevent blood loss but also provide key signals for matrix architecture reconstruction and for the recruitment, proliferation, survival and differentiation of cells endowed with new tissue formation, such as fibroblasts, smooth muscle cells and tissue-specific progenitors cells [113]. This is remarkably illustrated by the requirement of platelets in liver regeneration [114]. PMPs are also thought to contribute to vascular repair [115]. Hence, platelet activation is both necessary to tissue integrity and undesirable as it generates tissue-damaging signals. The complex network of signals that organize this fine-tuned equilibrium is only recently being biochemically dissected [55, 104]. Many questions remain on this balanced platelet friend or foe contribution, although they are of key importance to the pathophysiology of microvascular dysfunctions, such as in sepsis [68].

## Platelets contribute to the innate immune response against infection

The role of platelets in the defence against infection is increasingly stressed [83, 116]. Platelets are now acknowledged as *bona fide* pathogen sensors interacting directly or indirectly with a number of bacterial, viral, fungal and protozoan pathogens and their products, contributing to their clearance. Platelet interaction with bacteria depends on the nature and concentration of bacteria, interaction time, and involves multiple mechanisms. Toll like receptors-dependent and independent mechanisms, such as those involving Fcγ receptors, complement receptors or glycoproteins GPIIb-IIIa and GPIbα, contribute to platelet–bacteria interactions. Indirect interactions are also involved, such as via the binding of plasma proteins, including fibrinogen, von Willebrand factor, complement proteins or IgG, that bridge pathogens and platelets or via interaction with bacterial toxins. Interaction with pathogens can lead to platelet adhesion or to their activation, aggregation and release of platelet mediators [83, 117–120]. Mechanisms of pathogen clearance by platelets may be direct, through the release of various antimicrobial peptides and indirect via the release of platelet-derived mediators that coordinate chemotaxis and activation of immune cells [83, 116–118, 120, 121]. Infection is commonly associated with tissue injury. Injured and dying cells generate mediators such as alarmins that fuel inflammation [122]. Mediators generated by cell damage such as complement activation products and histones can activate platelets [123, 124]. Notwithstanding, platelets also contribute to the adaptive immune response to infection [17, 18, 22, 125].

The aforementioned role of platelets in the defence against pathogens suggests that they can interfere with the progression of infection. How can these observations be translated to sepsis pathophysiology [120, 124, 126]? Models suggest a protective role of platelets, as, for example, in streptococcal endocarditis, malaria or gram-negative pneumonia [127–129], and thrombocytopenia could be a risk factor for bacterial or fungal infection. Alternatively, platelets could contribute to spreading infection, via the transport of pathogens [130].

## Platelets in MOD pathophysiology

### Endothelium in MOD: a common pathophysiological denominator

**Fig. 3:**
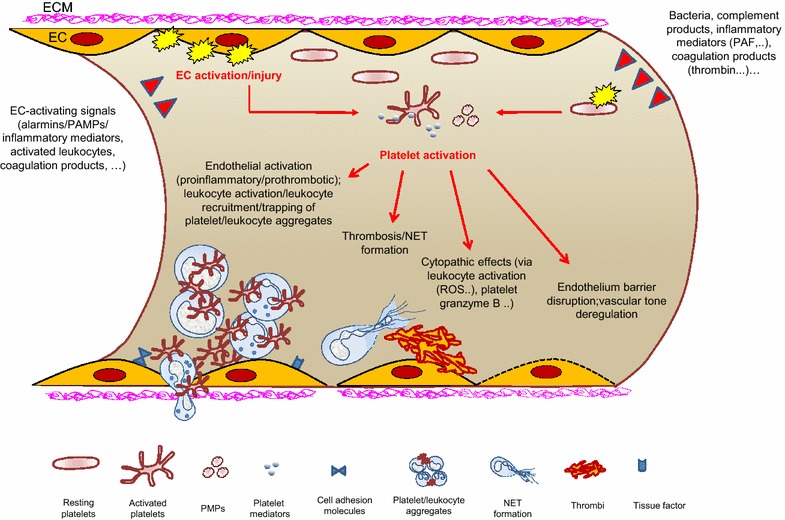
Microvasculature is a critical target of platelet activation in sepsis. Platelets circulate more concentrated close to the vascular wall and sense endothelium disturbances. In sepsis, many cellular and soluble actors concur to activate the endothelium. Activated/injured endothelium is a key driver of platelet activation. Signals generated by infection, inflammation and coagulation can also activate platelets in sepsis. The relative importance of platelet activation by PAMPs in sepsis is not well established. Platelet activation contributes to fuel various pro-inflammatory and pro-coagulant pathways with potential deleterious consequences on endothelium homoeostasis and integrity. Unmitigated platelet activation in sepsis may take a significant part in the complex global scenario that leads to impairment of the endothelium barrier and microcirculatory failure, a leading cause of organ dysfunction in sepsis. Only some pathophysiological events are schematized (see text for details). Scales are arbitrary. *ROS* reactive oxygen species, *EC* endothelial cells, *NET* neutrophils extracellular traps, *PMPs* platelet microparticles, *ECM* extracellular matrix

The pathophysiology of sepsis and its complications remains uncertain as much caution has to be applied in extrapolating to clinical sepsis results obtained in rodent models which have their own inherent complexities [131–134]. Within these extrapolation limits, experimental models have, however, yielded significant knowledge. Numerous studies have emphasized the orchestrating role of endothelium in sepsis, and endothelium injury could be one of the *primum movens* pathophysiological events in sepsis complications [102, 135–148]. Markers of endothelium injury are elevated in sepsis patients, although variably associated with sepsis severity [29, 32, 149]. Inflammation, thrombosis, capillary perfusion alterations are among key features of MOD microvascular alterations [102, 136, 150, 151]. Platelet activation can be detected in sepsis patients and sepsis models, and studies reported association with sepsis severity [79, 124, 152, 153]. Many signals can activate ECs and platelets in sepsis, including pathogens and mediators generated by inflammation and coagulation. Activated platelets may thus contribute to MOD via their role in inflammation and coagulation (Fig. 3) [23, 56, 83, 124, 141, 144, 152, 154].

### Platelets in acute lung injury (ALI) in sepsis

There are arguments to involve platelets in ALI pathophysiology [155–158]. Dysregulated inflammation and coagulation are central pathophysiological events in ALI and lung vascular endothelium injury is a primary cause of the alteration of the alveolar-capillary barrier leading to pulmonary oedema [159] [160]. Platelets are sequestered early in lung microvascular beds in ALI models and may contribute to the initial insult of lung endothelium [155, 161–164]. EC activation/injury by inflammatory stimuli, PAMPs and alarmins can generate signals mediating platelet accumulation and activation. Entrapment and activation of platelets in pulmonary capillaries will consequently feed the deleterious cascade of pro-inflammatory and pro-coagulant events in the lung [71, 156, 158, 159, 165]. Platelets can also induce apoptosis in the lung in sepsis models [166]. Among these events, platelet/neutrophil interactions have received considerable attention. Neutrophils are of critical importance in MOD, neutrophil influx being a hallmark of ALI and their inappropriate activation leading to tissue damage signals [167]. Platelets play an important role in neutrophil recruitment and activation in the lung, and platelet-mediated neutrophil activation results in the release of cytokines, chemokines, reactive oxygen species and NET generation [71]. Indeed, experimental models highlight the deleterious role of platelet/neutrophil and also platelet/monocyte interactions in the alteration of the alveolar-capillary integrity [71, 108, 168]. Coagulation activation and alveolar fibrin deposition are common findings in ALI, and platelets are thought to be key contributors to the dysregulation of coagulation in ALI, through their role in coagulation and NET generation [169]. Therefore, several studies suggest a platelet involvement in ALI. In fact, platelet depletion, blocking of platelet/neutrophil interaction, NET dismantling or antiplatelet treatments are protective in experimental models [96, 162–164, 170]. Although in vitro experiments show pro-inflammatory and pro-coagulant effects of PMPs, there is little evidence for a specific deleterious role of PMPs in ALI, a study difficult to address due to microparticle identification uncertainties and to the simultaneous presence of microparticles from various origins with heterogeneous functions [158, 171].

Increased vascular permeability is the basis for oedema in inflammation. The concept that platelets protect the basal barrier of alveolar capillaries is supported by experimental evidences, and thrombocytopenia put the lung capillary integrity at risk, particularly in inflammatory conditions. Indeed, severe thrombocytopenia results in increased alveolar-capillary permeability [71, 107, 108]. However, as mentioned above, platelet activation in inflammation can also disrupt endothelium barrier integrity, and platelet depletion is protective in several ALI models [69, 71, 162, 172]. How this dual endothelial barrier-stabilizing versus barrier-destabilizing property of platelets is organized and contribute to ALI progression, as well as the specific role of PMPs, is not understood. Such a differential effect of platelets in controlling endothelial barrier integrity is likely to be based on a complex balance between characteristics of inflammation in vascular beds, early or late phase, magnitude, role of other inflammatory players, i.e. leukocytes, and experimental models used. The changing relative importance during sepsis progression of platelet-activating signals, platelet count and proteome, interactions with leucocytes and ECs, underline the difficulty to dissect these mechanisms [69, 71, 76, 108, 173]. Moreover, platelets may play a positive role in the control and resolution of inflammation in lung injury, a mechanism that is only recently being understood [158]. The genetic background also plays a role in ALI-associated mortality and morbidity [174]. Platelet count is determined by genetic factors, and genetic studies point to an association between low platelet count and acute respiratory distress syndrome (ARDS) risk. Genetic variants within the LRRC16A locus (6p22) are associated with a low platelet count. Interestingly, a low platelet count links a single nucleotide polymorphism within this locus to ARDS risk [175].

### Platelets and acute kidney injury (AKI) in sepsis

Acute kidney injury (AKI), a major sepsis complication, is accompanied by hemodynamic disturbances such as decreased glomerular filtration rate and microcirculation alterations [146, 176–180]. The extent of apoptosis and necrosis in tubular lesions is debated. Subtle, heterogeneous, potentially reversible, cytopathic and adaptive cellular events (metabolic changes, mitochondrial dysfunction, autophagy, cell cycle arrest, etc.) may characterize tubular lesions in sepsis AKI [181–184]. Beyond the classic paradigm of renal hypoperfusion, the role of immune response pathways and particularly inflammation in AKI progression is increasingly stressed [1, 178, 185–195]. Alarmins, PAMPs, inflammatory mediators and  leukocytes can activate ECs in the renal microcirculation bed, leading to inflammation/thrombosis, metabolic alterations, oxidative stress, concurring to microvascular dysfunction. Due to the close dependence between TECs and tubular microvascularization, compromise blood flow and inflammation in the microvascular beds can lead to tubular epithelial cells (TECs) injury, driving inflammation, mitochondrial/metabolic alterations and various adaptive responses, including cell cycle arrest. Alarmins, PAMPs and inflammatory mediators may also impact TECs after being filtered, and TECs are active participants in kidney inflammation [192, 196–198].

In the highly vascularized kidney, platelet/endothelium interactions can be postulated to be of specific importance. In an AKI model in which selective kidney endothelial injury is realized, there are evidences for platelet contribution [199]. Platelets will be arrested and activated on the kidney endothelium activated by circulating deleterious signals. Inflammation-mediated alteration of EC glycocalyx can also favour platelet adhesion [141, 146, 200–203]. Platelets can also be activated by ischaemic blood flow disturbances in the septic kidney. Therefore, and although much remains to be understood, platelets may be pathophysiological players in sepsis AKI. On the other hand, as mentioned above, platelets contribute to the resolution of inflammation and vasculature integrity. Important questions remain with reference to the identification of soluble and cellular effectors that contribute to the resolution of inflammation and tubular regeneration in the kidney [204]. Microparticles, and PMPs more specifically, are elevated in sepsis and sepsis complicated by AKI [101, 102, 193]. However, their specific role remains to be addressed.

### Platelets and organ-to-organ crosstalk in sepsis

Despite the importance of the deleterious organ-to-organ communication in sepsis, underlying mechanisms are only beginning to be unravelled. Inflammatory signals are implicated in these communications [205]. Can platelets vectorize the exchange of pro-inflammatory and/or pro-coagulant signals that link injuries in distant organs? Interestingly, the activation of platelets at remote sites may mediate lung injury, as shown in mesenteric ischaemia/reperfusion models [206]. Platelets can mediate remote kidney damage induced by pneumonia [207]. Among platelet-derived mediators that could convey such a deleterious action, platelet factor 4 (CXCL4) and CD154 have been identified [208, 209]. When activated, platelets express CD154 and release a soluble form of CD154 [22, 210]; CD154 may bear a particular responsibility as, for example, the CD154/CD40 dyad plays a deleterious role in ALI, including pancreatitis-associated lung injury [211, 212], and as it could be brought to lung microcirculation via PMPs. Further, platelet CD154 mediates neutrophil recruitment in septic lung injury [213]. Although these results suggest a role for platelets, the extent and relative contribution of platelets, platelet-derived mediators, PMPs or circulating platelet/leucocyte aggregates in conveying deleterious signals at distance in patients with sepsis is unknown.

## Platelet count in sepsis and the dilemma of platelet transfusion

### Platelet count and dynamics of platelet count as determinants of clinical outcome in sepsis patients

Thrombocytopenia is common in sepsis and more generally in critically ill patients and has long been recognized as an independent risk factor for mortality in ICU patients and a sensitive marker for disease severity; the severity of sepsis is a risk factor for thrombocytopenia [6, 8–15, 214–221]. For these reasons, the platelet count is included in the ICU severity of illness scoring system. Platelet count kinetics is often biphasic in ICU patients, characterized by a moderate initial decrease in the first days followed by a rise [11, 216, 222]. Early thrombocytopenia and new-onset thrombocytopenia during ICU hospitalization are associated with a poor prognostic; the magnitude and duration of thrombocytopenia and the absence of relative increase in the platelet count have been linked to the poor outcome [6, 9, 11, 216, 221–227]. In a large recent study, which included 931 sepsis patients, a low platelet count at admission in the ICU was associated with an increased mortality risk [29]. Notably, patients with low platelet counts were more severely ill at ICU admission. Understanding pathophysiological links between platelet count alterations and clinical outcomes is therefore an important issue for the intensive care physician.

### The multiple causes of thrombocytopenia in sepsis patients

The association between thrombocytopenia and clinical outcome does not establish causality, and identifying the causes of thrombocytopenia is essential to patient management. Management of the underlying condition is a primary focus, and an important issue is platelet transfusion. Platelet transfusion may be ineffectual and deleterious in patients with, for example, intravascular platelet activation and have their own risks [228–230]. In a recent report, sepsis was identified as associated with ineffectual platelet transfusion, as evaluated by inadequate platelet count increase [231].

Several mechanisms, acting alone or in combination, can be responsible for a low platelet count in sepsis, and all steps of platelet life may be concerned. Decreased platelet production in the bone marrow can result from pre-existing conditions or from the inhibitory effect of pathogen toxins, drugs or inflammatory mediators on haematopoiesis. Peripheral mechanisms are essential causes of thrombocytopenia [15, 214, 218, 227, 232, 233]. The reduction in platelet half-life and their consumption/destruction may be linked to the many events of platelet activation occurring in sepsis, intravascular coagulopathy and immune mechanisms. Drug-induced thrombocytopenia, hemophagocytosis, bleeding, hemodilution are also major explanatory factors. Laboratory artefact of pseudothrombocytopenia can be encountered, and assessing the reality of thrombocytopenia is an important point [230].

Systematic investigations with routinely available tests can help to delineate mechanisms of thrombocytopenia [218, 232, 234]. An early rise of reticulated platelets follows endotoxin administration in humans, and the percentage of immature platelet fraction that evaluates thrombopoietic rate could be a useful tool to witness early bone marrow reaction predicting sepsis development [80, 235]. Apart from altering platelet count, sepsis and sepsis medications can also result in platelet function defect, adding another pathophysiological interface [236, 237]. A detailed description of these mechanisms and diagnostic/management guidance has been excellently reviewed and is beyond the scope of the present work [154, 222, 230, 233, 238–242]. A difficulty in approaching thrombocytopenia and its management is related to the paradox of platelets being potentially both deleterious and beneficial during sepsis course. In a first point of view, platelet count reduction is related to sepsis via consumption mechanisms including pathogen and pathogen product-mediated activation, induction of apoptosis, lysis and increased phagocytic clearance. Acute infections often lead to thrombocytopenia [58], and bloodstream infection is associated with lower platelet counts [221]. Coagulopathy, particularly DIC, platelet sequestration by leucocytes and by inflammatory vascular beds are also commonly stressed mechanisms of thrombocytopenia. Through these mechanisms, platelets can be perceived as bystanders whose destruction is related to the severity of infection and to the characteristics of the host response to the infectious challenge. In that case, the use of platelet transfusion may be perceived as being detrimental, via the fuelling of inflammation and coagulation. On the other hand, platelets are active players in pathogen clearance, leading to the possibility that a low platelet count and platelet function alteration may first favour infection. Further, platelets also protect vascular integrity; hence, maintaining an adequate threshold of platelet count seems a necessary target to prevent bleeding. In fact, platelet transfusions are mostly used to prevent or treat bleeding [228]. Conciliating such a paradox of platelets being both deleterious and beneficial is a challenging point for platelet-targeted therapeutic interventions in sepsis.

## Can platelets represent therapeutic targets and diagnostic tools in sepsis?

The clinical management of sepsis remains a difficult challenge, and pathophysiological advances have not yet been translated into effective therapeutic protocols [2]. Notably, strategies to counteract the runaway pro-inflammatory state in sepsis, such as inhibition of specific inflammatory mediators, have given disappointing results [243]. However, current knowledge on sepsis pathophysiology, highlighting multiple humoral and cellular factors in the inappropriate inflammatory response to infection, suggests that therapies targeting a single mediator will not demonstrate effectiveness [41]. Additional complexity is linked to individual disease susceptibilities and medical comorbidities that would necessitate individual approaches. Accumulating evidence therefore speaks for an integrated approach of sepsis treatment based on a better knowledge of its natural history.

The recently described involvement of platelets at the crossroads of several immune response pathways has led to the assumption that platelets or platelet-derived effectors represent therapeutic targets in sepsis. Platelet activation can drive multiple inflammatory and coagulation pathways, and targeting platelets offer the theoretical perspective of targeting simultaneously several deleterious pathways. Although the clinical relevance of animal models has many drawbacks, it is of interest that platelet depletion, inhibition of platelet functions and antiplatelet drugs show protection in experimental ALI or AKI [124]. P2Y12 inhibitors reduce inflammatory and pro-thrombotic mechanisms after endotoxin administration in humans [244]. Several observational and retrospective clinical studies have shown that antiplatelet agents such as acetylsalicylic acid, platelet P2Y12 inhibitor clopidogrel or GPIIb/IIIa antagonists reduce mortality or complications in critically ill patients [245–255]. However, some studies are conflicting [249, 252, 256] (Table 1). There is therefore a strong need for large randomized controlled clinical trials to investigate the effects of antiplatelet therapy in sepsis. The complexity of such studies relates in part to the heterogeneity of sepsis patients in terms of nature of the causal germ, site and severity of infection, multiple comorbidities, gender, age and genetic background. There is also individual variability in the concentration of antiplatelet agents that efficiently inhibits platelet function. A defective response to clopidogrel or aspirin treatment may concern up to 30 or 40% individuals, respectively [257–259]. Also, antiplatelet treatments have differential effects on platelet functions. Platelets treated with aspirin can still be activated by strong agonists, such as thrombin or ADP. Hence, in a full-blown pro-inflammatory/pro-coagulant condition as met in sepsis, it remains to be determined whether platelet activation is efficiently inhibited by antiplatelet treatments. Platelets are an important blood reservoir of pro-inflammatory molecules and may contribute to the “cytokine storm” that characterizes sepsis. However, many cellular players, including leucocytes and EC, also produce such mediators, and the relative contribution of platelets is not understood. In a recent study, antiplatelet therapy did not significantly reduce plasma pro-inflammatory cytokines levels in sepsis patients [260]. Antiplatelet agents have also been shown to have indirect off-platelet effects, a mechanism which importance is not yet established [261]. Finally, the impairment of platelet function may have undesirable consequences, such as bleeding or the blunting of platelet protective functions.

**Table 1:** Summary of cohort studies on antiplatelet agents and sepsis

Authors	Study year	Study type and setting	Patient number	Antiplatelet agent	Patients	Study conclusions	Potential limitations
Wang et al. [268]	2016	Meta analysis of cohort studies	14,612	ASA, clopidogrel, ticlopidine	ICU patients with ARDS predisposing conditions	Reduced mortality and lower incidence of ARDS	Non-sepsis patients included Treatment bias of antiplatelet agents
Kor et al. [269]	2012–2014	Multicenter, double-blind, placebo-controlled, randomized clinical trial	390	ASA	Patients with elevated risk for developing ARDS in the emergency department	ASA did not reduce the risk of ARDS and 28-day or 1-year survival	Non-sepsis patients included Low rate of ARDS development
Wiewel et al. [260]	2011–2014	Prospective observational study with propensity matching	972	Mostly ASA	Sepsis within 24 h after admission in 2 mixed medical/surgical ICU	Antiplatelet therapy was not associated with alterations in the presentation or outcome of sepsis or the host response	Treatment bias of ASA Inadequate patient number and power
Osthoff et al. [270]	2001–2013	Retrospective cohort study with propensity matching	689	ASA	Patients with *S. aureus* and *E. coli* bloodstream infection admitted in a single medical/surgical ICU	Low-dose ASA at the time of bloodstream infection was strongly associated with a reduced short-term mortality in patients with *S. aureus* bloodstream infection	Treatment bias of ASA at the time of enrolment Severity at presentation was not included in the analysis model Inadequate patient number and power
Tsai et al. [255]	2000–2010	A nation-wide population-based cohort and nested case–control study	683,421	ASA, clopidogrel, ticlopidine	Sepsis	Antiplatelet agents were associated with a survival benefit in sepsis patients	Claims database
Chen et al. [253]	2006–2012	Secondary analysis of prospective cohort with propensity matching	1149	ASA	Patients admitted in a mixed ICU for at least 2 days	Decreased risk of ARDS	Non-sepsis patients included Treatment bias of ASA
Boyle et al. [271]	2010–2012	Prospective observational study	202	ASA	ICU patients requiring invasive mechanical ventilation	Reduced risk of ICU mortality	Treatment bias of ASA Non-sepsis patients included
Valerio-Rojas et al. [249]	2007–2009	Retrospective cohort with propensity matching	651	ASA, clopidogrel	ICU patients with sepsis	No decrease in hospital mortality but decreased incidence of ARDS	Inadequate patient number and power Unmeasured bias and confounding
Otto et al. [251]	2013	Retrospective cohort	886	ASA, clopidogrel	Surgical ICU patients with sepsis and a minimum length of stay of 48 h and a history of atherosclerotic vascular diseases	ASA treatment reduced the ICU and hospital mortality. Combination of ASA with clopidogrel did not show any significant effect on mortality. Clopidogrel alone might have a similar benefit	Unmeasured bias and confounding
Sossdorf et al. [250]	2013	Retrospective cohort	979	ASA	Septic patients admitted to a surgical ICU	Decreased mortality with ASA or NSAIDs was associated with decreased hospital mortality. No benefit when ASA and NSAIDs are given together	Unmeasured bias and confounding
Eisen et al. [248]	2000–2009	Retrospective cohort study with propensity matching	7945	ASA	ICU patients with SIRS/sepsis on ASA at the time of SIRS/sepsis	ASA was associated with survival	Treatment bias of ASA at the time of enrolment and confounders
O’Neal et al. [272]	2006–2008	Cross-sectional analysis of a prospective cohort	575	ASA and Statin	Patients admitted in a mixed ICU for at least 2 days	ASA was not associated with the diagnosis of ALI/ARDS, sepsis or hospital mortality	Treatment bias of ASA Unmeasured bias and confounding Non-sepsis patients included
Erlich et al. [246]	2006	Retrospective cohort	161	ASA, clopidogrel, ticlopidine	Adult patients admitted in a medical ICU with a major risk factor for ALI	Reduced incidence of ALI/ARDS	Treatment bias of ASA Non-sepsis patients included
Kor et al. [256]	2009	Second analysis of prospective multicenter observational study	3855	ASA	Consecutive, adult, non-surgical patients with at least one major risk factor for ALI	ASA was not associated with ICU or hospital mortality and ICU or hospital lengths	Treatment bias of ASA Non-sepsis patients included Unmeasured bias and confounding
Storey et al. [273]	2006–2008	Post hoc analysis PLATO study	18,421	Ticagrelor vs clopidogrel	Patients with acute coronary syndrome	Reduced mortality following pulmonary infection and sepsis in acute coronary syndrome with ticagrelor	Unmeasured bias and confounding
Winning et al. [245]	2007–2009	Retrospective cohort	615	ASA, clopidogrel	Consecutive patients admitted in a mixed ICU	Reduction in organ failure and mortality in critically ill patients with pre-existing medication	Non-sepsis patients included Treatment bias of ASA
Winning et al. [274]	2002–2007	Retrospective cohort	224	ASA, clopidogrel ticlopidine	Patients admitted for CAP not receiving statins and using antiplatelet drugs for more than 6 months	Reduction in need of intensive care treatment and length of hospital stay	Unmeasured bias and confounding
Gross et al. [275]	2001–2005	Retrospective cohort	417,648	Clopidogrel	All adult (≥ 18 years) Medicaid beneficiaries in Kentucky	Increased CAP incidence and no significant reduction in severity	Claims database

*ASA* Acetylsalicylic acid, *ARDS* acute respiratory distress syndrome, *ALI* acute lung injury, *CAP* community-acquired pneumonia, *NSAID* non-steroidal anti-inflammatory drug

As mentioned above, elucidating mechanisms of thrombocytopenia in sepsis are essential with reference to transfusion. Platelet transfusion is mostly used to prevent/treat bleeding [228, 229]. The risk of bleeding increases with the severity of thrombocytopenia [222]. The threshold of platelet count ensuring protection may be higher in sepsis patients, reflecting the severity of the disturbance of the vascular beds. Commonly advocated threshold of platelet count is in the range of 10–50 × 10^9^/L, depending on clinical situations, additional bleeding risks, evidence for central thrombocytopenia. The risk of bleeding is, however, not straightforwardly linked to the depth of thrombocytopenia, in the context of a sustained production of platelets, and additional parameters in the critically ill patient may interfere; indeed, the risk of bleeding is also increased for platelet counts between 50 and 100 × 10^9^/L [8, 9, 222, 229]. In fact, there is a poor evidence-based clinical benefit of platelet transfusion in the non-bleeding ICU patient [154, 228–230, 242]. The lack of a clear understanding of thrombocytopenia causes makes the risk/benefit assessment difficult, as there is a theoretical risk to aggravate the underlying pathophysiology [229]. The main regulator of platelet production, TPO, is elevated in sepsis and related to the platelet count [262, 263], which may be linked to the reduction in platelet mass or stimulation of TPO production by inflammatory mediators. Experimental models show that TPO neutralization reduces the severity of organ damage [264]. However, in the clinics, the potential benefit of TPO administration in thrombocytopenic patients in sepsis has been recently suggested [265]. At this stage, results from randomized controlled trials remain necessary to evaluate TPO interest in sepsis.

If the interpretation of thrombocytopenia in sepsis patients is made difficult by the multiplicity of underlying mechanisms, the platelet count by itself may hold valuable information [242]. The platelet count may represent a surrogate marker of the severity of organ dysfunction. A low platelet count occurring even early in sepsis patients is indeed recognized as a sign of poor prognostic; however, a single platelet count at admission may have little pertinence [266], and the kinetics of platelet counts appears to have a deeper meaning. Two alterations of this kinetics have been shown to be of clinical interest in sepsis patients, suggesting that they must be given specific attention. Both the magnitude of the drop in platelet count rather that thrombocytopenia per se, and the non-resolution of thrombocytopenia are strong predictors of mortality in sepsis [9, 15, 267]. The onset and dynamics of thrombocytopenia have been stressed as potential diagnostic approaches in ICU patients [222].

## Conclusion

Platelets play key roles in various aspects of the immune response, suggesting that they take a significant part in sepsis pathophysiology. Therapeutic control of platelet functions would offer the perspective of targeting simultaneously several deleterious pathways in sepsis. The difficult extrapolation of experimental models to clinical sepsis and the conflicting results of clinical studies do not allow us today to introduce an antiplatelet agent in clinical practice. However, septic critically ill patients treated with long-term antiplatelet agent may benefit from the continuation of their treatment in the absence of bleeding risk, avoiding a rebound of platelet reactivity. The multiple facets of platelet involvement in sepsis therefore represent substantial challenges to the clinician and call for a deeper understanding of the relative importance of platelet contribution to determine their ultimate clinical significance.

## Abbreviations

ALI: acute lung injury
ARDS: acute respiratory distress syndrome
PAMPs: pathogen-associated molecular patterns
DIC: disseminated intravascular coagulation
EC: endothelial cell
ICU: intensive care unit
MOD: multi-organ dysfunction
PMPs: platelet microparticles
SIRS: systemic inflammatory response syndrome
TEC: tubular epithelial cell
AKI: acute kidney injury
ECM: extracellular matrix
NET: neutrophil extracellular traps
TPO: thrombopoietin
TREM-1: triggering receptor expressed on myeloid cells 1
