# Correction to: Blood platelets and sepsis pathophysiology: A new therapeutic prospect in critically ill patients?

**DOI:** 10.1186/s13613-018-0378-6

**Published:** 2018-02-28

**Authors:** Antoine Dewitte, Sébastien Lepreux, Julien Villeneuve, Claire Rigothier, Christian Combe, Alexandre Ouattara, Jean Ripoche

**Affiliations:** 1 INSERM U1026, BioTis, Univ. Bordeaux, 33000 Bordeaux, France; 2 Department of Anaesthesia and Critical Care II, Magellan Medico-Surgical Center, CHU Bordeaux, 33000 Bordeaux, France; 3 Department of Pathology, CHU Bordeaux, 33000 Bordeaux, France; 4 Cell and Developmental Biology Department, Centre for Genomic Regulation, The Barcelona Institute for Science and Technology, 08003 Barcelona, Spain; 5 Department of Nephrology, Transplantation and Haemodialysis, CHU Bordeaux, 33000 Bordeaux, France; 6 INSERM U1034, Biology of Cardiovascular Diseases, Univ. Bordeaux, 33600 Pessac, France

## Correction to: Ann. Intensive Care (2017) 7:115 10.1186/s13613-017-0337-7

Upon publication of the original article [1], it was noticed that the title was incorrect. Instead of ‘critical’, it should read ‘critically’, and therefore, the correct title should be:

Blood platelets and sepsis pathophysiology: A new therapeutic prospect in critically ill patients?

Furthermore, the word ‘liver’ on page 5, line 38 of the PDF, in the sentence ‘This is remarkably illustrated by the requirement of platelets in liver regeneration’ should in fact be ‘lung’.
